# Engaging a community to enable disease-centric data sharing with the NF Data Portal

**DOI:** 10.1038/s41597-019-0317-x

**Published:** 2019-12-13

**Authors:** Robert J. Allaway, Salvatore La Rosa, Sharad Verma, Lara Mangravite, Justin Guinney, Jaishri Blakeley, Annette Bakker, Sara J. C. Gosline

**Affiliations:** 1 Sage Bionetworks, Seattle, WA 98146 United States; 2 Children’s Tumor Foundation, New York, NY 10017 United States; 3 Department of Neurology, The Johns Hopkins School of Medicine, The Neurofibromatosis Therapeutic Acceleration Program, Baltimore, MD 21287 United States

**Keywords:** Data acquisition, Research management

## Abstract

A significant challenge facing rare disease communities is finding a sufficient quantity and variety of data to develop and test disease-specific hypotheses. Here we describe an approach to data sharing in which stakeholders from the neurofibromatosis (NF) research community collaborated to develop a disease-focused data portal with the goal of supporting scientists within and outside the community as well as clinicians and patient advocates.

Neurofibromatosis (NF) refers to a family of rare neurogenetic diseases characterized by disfiguring tumors, cancers, and pain among other symptoms. These include neurofibromatosis type 1 (estimated birth incidence of 1:3000), neurofibromatosis type 2 (estimated birth incidence of 1:25000), and schwannomatosis (estimated birth incidence 1:40000). The diversity of phenotypes of NF and rarity of the diseases are factors which have contributed to the reduced the pace of therapeutic discovery. One reason for this is that gathering data to develop and test new scientific hypotheses is challenging. While many scientists have generated model systems and data to study NF, a paucity of comprehensive and public NF data was identified as a key impediment by two NF funders: the Children’s Tumor Foundation (CTF), and the Neurofibromatosis Therapeutic Acceleration Program at Johns Hopkins (NTAP). To address this, these two foundations joined forces with Sage Bionetworks to form the NF Open Science Initiative (NF-OSI) which is a community to support the sharing of data, resources, and tools with a goal of accelerating the pace of research for the NF community. Building a community around open science has required engagement from many stakeholders: the funding agencies, the scientists conducting research, and scientists focused on data curation, storage, and sharing. Here we describe the roles of each of these stakeholders has in fulfilling the NF-OSI goal of open science^1,2^ and how this effort resulted in the creation of the NF Data Portal.

## Roles of the NF Research Community Stakeholders

To promote an open science model for the NF community, CTF (in 2014) and NTAP (in 2015) started including a data sharing requirement in their research funding agreements. This was initially implemented in the CTF Synodos program, a multi-institutional multi-investigator award, to help all of the project collaborators to internally share and access the data through a common workspace. This requirement stipulated that researchers would publicly share all study-related data upon completion of the project. Specifically, both CTF and NTAP required that the project-related datasets would be uploaded on the Synapse collaborative science platform (www.synapse.org) as they were generated and shared after the study was complete^3^. After the completion of the project, passage of an embargo period (up to 18 months), and permission of the primary investigator, the uploaded results were made publicly available. Members of these funding organizations worked closely with grant recipients to ensure that data was being uploaded in a manner that considers timeliness and utility of the data to the broader scientific community while also allowing the primary investigator an opportunity to finalize their research.

NF-OSI funded researchers were required to regularly upload datasets generated by their studies. By mid 2019, over 61 public and embargoed NF1, NF2, and schwannomatosis studies were available on Synapse. The nature of the associated data ranged broadly in both scope and nature, as they included discovery research (genomic variants, gene expression, proteomics, kinomics, chromatin regulation, cellular physiology, low and high throughput drug screening), translational studies data (pharmacokinetics, tumor volumes, imaging), and clinical data (patient-reported outcome metrics and clinical survey data). In addition to data, these researchers have generated tools, publications, and other outputs that could be linked back to the original studies.

To allow others to understand the nature of and reuse the scientific outputs that were shared, scientists focused on curating the data worked closely with all NF-OSI investigators to ensure that each dataset was uploaded and annotated using a standard metadata schema. This schema was consistent across all NF-OSI projects, and leveraged community metadata standards like the NCI Thesaurus and other ontologies^4,5^ as necessary to describe the data. These scientists also helped to manage data access permissions to enable the organization and preparation of the data ahead of data release, and also to ensure that data was restricted to approved users if the data were sensitive (e.g. patient data). These access restrictions are made available to the users so that they can identify what they are able to download. Finally, as an additional part of this curation effort, datasets, publications, and research tools were tracked, described, and annotated.

During the course of curating and analyzing research generated by CTF and NTAP-funded projects on the Synapse collaborative research platform, it became increasingly obvious to stakeholders in the NF-OSI that sharing diverse data across the community presented numerous challenges. The most apparent challenge was helping researchers discover & explore the data and the consequent datasets, tools, and publications. Sometimes, publications were used to point researchers to new NF datasets^6–9^, but this was not always the case. Additionally, the long latency between data production and publication was causing a gap in knowledge about research activities from ongoing or finalized but unpublished studies. Ultimately, the NF-OSI lacked a user-friendly place to explore studies and their content, a critical shortfall when considering the FAIR Guiding Principles^10^.

## Building a Gateway for Exploring NF Research

To help NF research stakeholders explore the NF-OSI studies and data, we decided to develop a data portal. This portal needed to be data-type agnostic to facilitate the discovery of all studies together with their associated datasets, files, tools, and publications. For data for which there are accepted standard repositories, the funders encourage deposition of data when appropriate repositories exist (for example, genomic data in repositories such as dbGaP). However, a large amount of the data we receive has no accepted standard repository (e.g. drug screening data, animal model data, imaging data). We hypothesized that providing these data in a common platform would increase their utility and findability, and that such a resource would complement data-specific repositories such as those for genomic data like the Gene Expression Omnibus (GEO)^2^ or the Sequence Read Archive (SRA)^1^. We additionally hypothesized that a portal specific for NF would allow more informed curation of content to ensure its relevance to the pertinent scientific questions in this domain. While many portals are well-designed for conveying large quantities of similar data, they are not generally designed to support a disease community in exploring diverse types of data specific to their disease. We opted for an alternative approach and instead designed a disease-focused but data-type-agnostic portal.

**Fig. 1:**
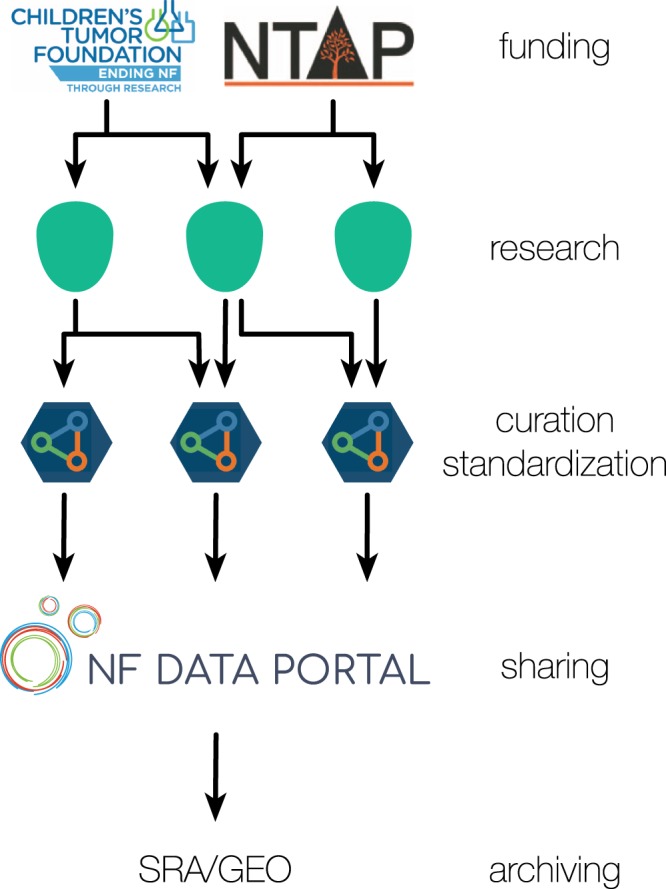
The relationship between the funding, research, curation, and sharing steps of the NF-OSI. CTF and NTAP fund researchers, who in turn generate datasets, tools, and publications. Data are uploaded to Synapse, where the projects are curated, standard metadata is applied to the files, and publications are tracked. After these steps, these projects are connected to the NF Data Portal to facilitate exploration of study files, datasets, tools, and publications. When the nature of the data permit (i.e. the data are public, non-sensitive, and of a format that is compatible with sequence/array-focused archives) the data are also deposited in public archives such as the Sequence Read Archive (SRA)^1^ or the Gene Expression Omnibus (GEO)^2^.

The NF Data Portal (http://nfdataportal.org, Fig. 1) was developed using a user-centered design approach to help all members of the NF community acquire the information they need at the appropriate level of detail. When a user arrives at the portal, they see a small visual summary of the content that can be explored in the portal as well as highlights of new studies, datasets, publications, and tools. Organization-specific pages show all projects and data coming from specific organizations and serve as a landing page for those organizations to highlight their grantees. The “Explore” page allows users to dive into the studies, files, datasets, as well as publications that have been produced using these data. Notably, we opted not to include publications for which linked data or studies are not available, as the scientific community uses PubMed as a standard method to access the majority of publications and we are not attempting to replace that service. In the NF Data Portal, users can explore the aforementioned research objects using the underlying metadata dictionary. All of the metadata exposed to the user has been selected for its relevance to the data as well as to the types of questions frequently asked by the NF community. Therefore, this approach is a flexible system that can be curated to the needs of the community and is primarily driven by the metadata instead of the data itself.

By building the NF Data Portal as a layer on top of Synapse, we were able to use the metadata and security management features without exposing the underlying complexity to users that are primarily interested in acquiring data that is relevant to them. When a user finds a dataset of interest, they can download the data from Synapse itself, where any applicable access restrictions are enforced. This approach allows users to rapidly explore the metadata of embargoed projects, allowing them to observe what projects exist and what data they are generating, while the actual data remains secure until the study is finalized and published, after which they can be easily made public. The public data release can be either funder-mandated or a dialogue between funder and researchers, so it can conform to a variety of open science funding models.

## Value of a Community-Oriented Data Portal

The increasing presence of scientific data portals, such as those developed for cancer and neurodegenerative disease underscores the importance of FAIR data sharing to support open science. Some examples of these include the Genomic Data Commons and the Clinical Proteomic Tumor Analysis Consortium Portal in cancer, the KidsFirst Data Resource Center in pediatric cancer, and the AMP-AD Portal in Alzheimer’s. The development of the NF Data Portal, driven by NF-focused researchers and organizations, showcases a unique approach to portal development that we believe could serve as a model for other communities. We designed the portal experience to allow researchers from different backgrounds (e.g. experimental, computational) to find research relevant to their scientific questions. The focus on a single family of syndromes enabled us to expand the scope of data types. This more focused approach to curation has spurred independent members of the NF research community to contribute related studies, data, and tools that may not have been specifically funded by the explicit members of the NF-OSI. This targeting also allowed us to conduct user research within a smaller set of users to converge on a focused research exploration experience. The portal has also helped introduce concepts such as metadata management and data structures to a community that is unfamiliar with it, make rare disease data accessible to data scientists, and help researchers and funders identify gaps in publicly available-disease specific datasets. In addition to helping bring the research community together, the portal has helped to grow the NF-OSI and expand the coverage of the portal by encouraging additional funding organizations, such as the Congressionally Directed Neurofibromatosis Research Program (CDMRP NFRP) and Neurofibromatosis Research Initiative (NFRI), to participate^11^.

Beyond encouraging new funding partners to participate in the NF-OSI, we have several specific plans to improve the utility of the portal. Active areas of improvement include better documentation and tooling for those that wish to contribute new or historical NF data, adoption of common metadata standards to make NF research objects more searchable (e.g. implementing schema.org Dataset markup), better communication of data governance-imposed access requirements in the portal, improved dataset attribution to better credit data contributors, clearer dashboarding to summarize available data, and easier-to-use licensing of data for contributors.

We are also exploring options to handle the challenge of long-term data preservation, which appears to be an unsolved challenge for many, if not all, biomedical data repositories. However, we hypothesize that solutions like deposition of data in multiple repositories, aligning with federally-led initiatives for long-term data storage, and engaging additional funders to support the NF-OSI may be solutions to aid in the long-term availability of this data. In addition, when non-restricted sequence or array-based data are released from embargo, we deposit the data in public archives such as SRA^1^ or GEO^2^ to ensure its continued availability in standard data-specific repositories (e.g. SRA project SRP125359).

Finally, an ongoing challenge for this initiative is expanding its coverage to ensure that the portal is capturing useful quantities of community-generated data. While some recently-added studies were contributed voluntarily and not as a consequence of a funding requirement, the vast majority of data contributions are funder-mandated. Therefore, we are not only working to engage new NF funders to help collect data, but also exploring options for retrospective collection and harmonization of NF data from public repositories such as GEO and SRA. We are also using other approaches to encourage the contribution of additional data such as organizing data-oriented community outreach events like hackathons.

Based on our experiences working with the NF community, we advocate that a data-agnostic and disease-centric approach to open science may be particularly useful in under-served or rare disease research communities. This approach can serve as a model for other small disease communities for which data and samples are scarce, as this community-centric data portal ties together disparate data into larger, more quantifiable, datasets. The role of funders (both private and federal) in mandating and supporting data sharing and data curators in annotating and indexing datasets are two pivotal elements to incentivize researchers to share their data and create a useful and reliable community resource. We posit that the ability to flexibly build data portals by and for a specific disease community will further focus collaborative efforts, fuel scientific discoveries, and foster open science as a standard practice in rare disease communities.
